# Two-Stream Mixed Convolutional Neural Network for American Sign Language Recognition

**DOI:** 10.3390/s22165959

**Published:** 2022-08-09

**Authors:** Ying Ma, Tianpei Xu, Kangchul Kim

**Affiliations:** Department of Computer Engineering, Chonnam National University, Yeosu 59626, Korea

**Keywords:** ASL image recognition, two-stream, correlation information, CNN

## Abstract

The Convolutional Neural Network (CNN) has demonstrated excellent performance in image recognition and has brought new opportunities for sign language recognition. However, the features undergo many nonlinear transformations while performing the convolutional operation and the traditional CNN models are insufficient in dealing with the correlation between images. In American Sign Language (ASL) recognition, J and Z with moving gestures bring recognition challenges. This paper proposes a novel Two-Stream Mixed (TSM) method with feature extraction and fusion operation to improve the correlation of feature expression between two time-consecutive images for the dynamic gestures. The proposed TSM-CNN system is composed of preprocessing, the TSM block, and CNN classifiers. Two consecutive images in the dynamic gesture are used as inputs of streams, and resizing, transformation, and augmentation are carried out in the preprocessing stage. The fusion feature map obtained by addition and concatenation in the TSM block is used as inputs of the classifiers. Finally, a classifier classifies images. The TSM-CNN model with the highest performance scores depending on three concatenation methods is selected as the definitive recognition model for ASL recognition. We design 4 CNN models with TSM: TSM-LeNet, TSM-AlexNet, TSM-ResNet18, and TSM-ResNet50. The experimental results show that the CNN models with the TSM are better than models without TSM. The TSM-ResNet50 has the best accuracy of 97.57% for MNIST and ASL datasets and is able to be applied to a RGB image sensing system for hearing-impaired people.

## 1. Introduction

According to the World Federation of the Deaf (WFD) [1], sign languages are used by about 70 million deaf people worldwide. Sign recognition could assist the hearing-impaired and normal people break down social barriers. Because American Sign Language (ASL) is simple and valuable among all sign languages, it has been used for name spelling, book spelling, and letter correction as the primary language of hearing-impaired people in North America [2]. Thus, ASL is indispensable for hearing-impaired people. However, ASL is always used as a complementary language for alphabetic spelling in uncommon situations, and the improvement of the ASL recognition system is often overlooked. Although most communication technologies developed can well support spoken or written language translation, they are still insufficient for ASL. Therefore, building an accurate ASL recognition model is necessary to communicate better as an assistant tool for hearing-impaired people in name spelling, book spelling, and letter correction.

Some deep learning techniques have been used for sign language recognition [3]. Among these methods, the Convolutional Neural Network (CNN) usually achieves better recognition accuracy in sign language. A single-stream CNN always uses one or a group of images for recognition. A convolution kernel operates on each independent image separately [4].

It is difficult for a single-stream CNN to obtain characteristics of the relationship between related images during the training process, which brings challenges to CNN in dynamic gesture recognition. With CNN development, a two-stream structure appears to allow the CNN model to obtain multiple features for more accurate computation [5]. Undeniably, the two-stream structure brings opportunities for more accurate recognition of dynamic sign language.

In the two-stream CNN structure, the premise of obtaining different features for more accurate computation is the task of ASL feature extraction from multiple deep models.

Because feature extraction and classification for ASL images are carried out by two different deep CNN structures and features are repeatedly computed too much, heavy computational tasks are required. In this paper, a Two-Stream Mixed (TSM) method is proposed to fuse ASL features using only one convolution layer, preventing features from being repeatedly computed. The fusion feature map prepared with TSM is then applied to a deep CNN model to calculate mixed features and obtain accurate classification results. The TSM is composed of addition and concatenation operations. The addition operation is used to enhance the expression of correlation information between images. Two sign language images are used as input of the TSM, then the feature maps are mixed by an addition operation (mixing feature maps). The concatenation operation aims to preserve the original image information. Feature maps of the original information (convolutional feature maps) are concatenated with feature maps of correlation information (fusion feature maps). Using the TSM method, useful features are extracted and used as inputs of classifiers. Models of LeNet [6], AlexNet [7], ResNet18 [8], and ResNet50 [9] with or without TSM are compared to evaluate the performance of the TSM. The model can be used in sensor-based application as a recognition part for hearing-impaired people to break the communication difficulty.

The contribution of the paper can be summarized as follows:

- A Two-Stream Mixed method (TSM) including addition and concatenation operations is proposed to achieve better feature extraction for ASL and to reduce computation burden.
- The proposed TSM is applied to deep neural networks for the static hand gesture language MNIST and ASL Alphabet dataset.
- Models of LeNet, AlexNet, ResNet18, and ResNet50 with TSM or without TSM are compared to evaluate the performance of the TSM method.
- The TSM-ResNet50 model can be used as a sensor of the ASL recognition system for hearing-impairing people.

This paper is organized as follows: Section 2 reviews the recent literature and explains problems in gesture recognition. Section 3 reviews several deep learning algorithms and introduces the proposed TSM method. Research results are explained in Section 4. The discussion between other methods and the proposed method is shown in Section 5. Finally, Section 6 provides conclusions.

## 2. Literature Review

Many papers related to sign languages recognition have been published recently to help hearing-impaired people. Adewuyi et al. combined electromyography data of fingers and arm muscles to classify handgrip and finger movements [10]. Huang et al. combined human hand acceleration, angular velocity, and muscle electrical data with the K-Nearest Neighbor (KNN) algorithm through a dual-channel method to recognize gestures [11]. In order to achieve better recognition results, some works used more than one piece of modal information, which is called a multi-modal method [12]. The Recurrent Neural Network (RNN) is a type of neural network used to process sequence data [13]. Cate et al. used RNN for time series modeling and recognized 95 types of sign language vocabulary [14]. Chai et al. proposed a dual-stream RNN network (2S-RNN) in 2016 [15]. The model could extract skeleton data and gradient histogram features as the input of another RNN network. This model ranked first in the CHALEARN Gesture Recognition Challenge. Li et al. proposed new hand type descriptors in 2017 and performed LSTM-based timing modeling on these descriptors, which achieved accurate recognition results in Chinese sign language recognition [16].

Lin et al. proposed a combination of a masked RES-C3D network and LSTM network in 2018 and achieved a 68.42% recognition accuracy on the chalearn dataset [17]. Pu et al. proposed a sign language recognition framework based on a three-dimensional residual network and dilated convolutional network in 2018 [18]. They also proposed an iterative optimization strategy based on the CTC algorithm. Wang et al. proposed a hybrid deep structure composed of time-domain convolution, a bidirectional recursive unit, and a fusion layer with an optimization method based on CTC loss [19]. However, this model is complex with high hardware requirements. Kopuklu et al. proposed a CNN recognition method that fuses motion information into static images, achieving good recognition results [20]. Devineau et al. proposed a CNN three-dimensional dynamic gesture recognition based on hand skeleton data. It uses convolution to process hand bone joints, achieving a high recognition accuracy [21]. Its disadvantage is that it has high hardware requirements for data collection.

Sign language recognition is still poor in practicality. Processing of dynamic gestures cannot be completely separated from higher hardware requirements. This situation makes sign language recognition development face a bottleneck. Some methods usually combine color information (RGB format), depth map information, and bone joint point information for dynamic gesture recognition. However, the acquisition of information except for RGB images usually requires a specific sensor, such as Microsoft’s Kinect, ASUS Xtion Pro, or Intel’s Realsense3. On the contrary, the gesture recognition technology based on RGB data has the advantages of convenient use and low cost [22]. In addition, it is easy to find surveillance cameras in many public spaces. Moreover, there are more interactive environments. This is also one of the reasons why people are committed to the development of using only RGB image data to recognize dynamic gestures. In addition, Vision transformer [23] and Tab transformer [24] have been successfully applied in image recognition. A transformer method based on sign language recognition has been proposed [25], where image frames from SL video are linearly embedded and the resulting sequence of vectors is fed back to a standard encoder to increase the model attention. However, these methods focus on dealing with complex and continuous sign language videos. The transformer method applied in a relatively simple expression of the ASL alphabet wastes too many computational resources.

**Figure 1:**
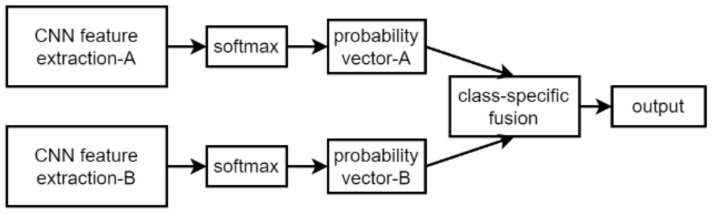
The 2S-CNN structure.

The human binocular visual system can inspire us. Richer image features can be extracted by the principle of optic chiasm in binocular vision cells. This has inspired the CNN to obtain better results in image recognition. A two-stream CNN [26,27] has achieved good results in the field of computer vision. Huang et al. proposed the LS-HAN network using a two-stream three-dimensional convolution neural network for sign language recognition and designed the impact of different loss functions on recognition [28]. QingGao et al. proposed a two-stream CNN model (2S-CNN) [29] using advantages of hand-gesture RGB and depth information by fusing these two kinds of information, as shown in Figure 1. One channel of 2S-CNN extracts features of ASL hand gestures. The other channel extracts 3D space features of gestures. Finally, outputs of these two channels are fused using a class-specific fusion method to achieve the final prediction. Although this method used for classification has achieved great success in a two-stream architecture, it also has shortcomings in motion information retention from dynamic gesture recognition.

Dynamic gestures are included in the composition of ASL. It is necessary to improve the recognition accuracy of dynamic motions for better application to ASL recognition. Therefore, a TSM-CNN model is proposed to increase the accuracy of ASL recognition, particularly in dynamic gestures.

## 3. Methodology

### 3.1. Datasets

Image datasets used in this paper included the static hand-gesture language MNIST dataset [30] and the ASL Alphabet dataset [31] from Kaggle’s website. All images in both datasets were captured by the camera sensor. Therefore, it can be verified by using these two datasets that the model proposed in this paper can be applied as a recognition part in a RGB capture-based translation tool for hearing-impaired people.

**Figure 2:**
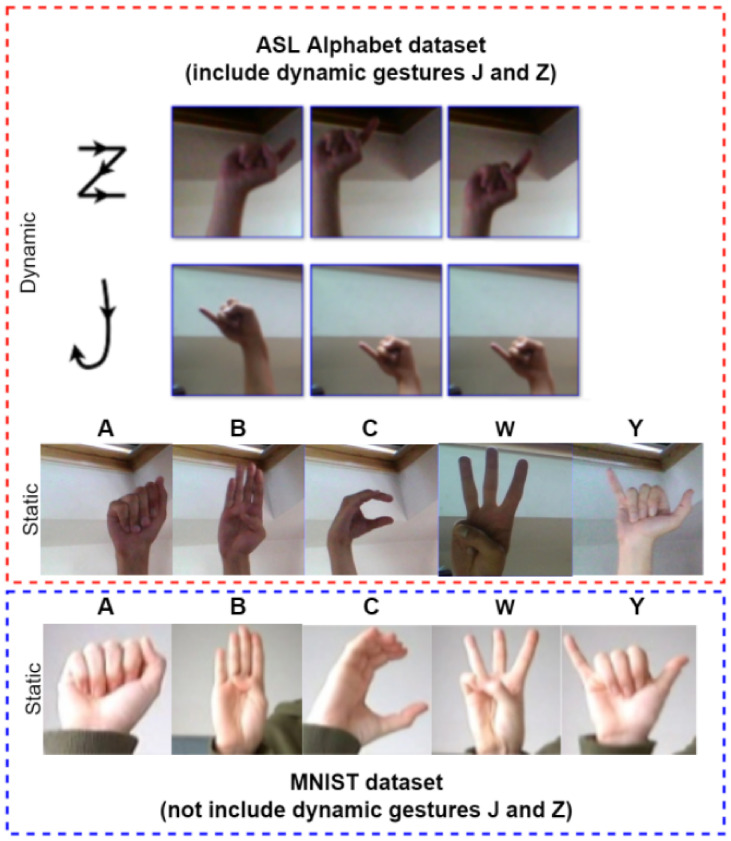
Gesture image examples in ASL Alphabet and MNIST datasets.

The image data examples are shown in Figure 2. ASL is a gesture language with a simple expression that mainly contains static and dynamic gestures. In “static” gestures, a gesture represents the meaning of an American letter, and the letters “J” and “Z” in ASL are expressed by moving gestures called “dynamic” gestures in this paper.

In the ASL Alphabet dataset, the image data contain 87,000 images. There are 29 classes, with each class having 3000 images. Of these 29 classes, 26 were captured for letters A-Z and three classes were captured for SPACE, DELETE, and NOTHING. In this dataset, 85% of the data were used for training and 15% were used for testing.

The MINIST dataset contains American sign language gestures from letters A to Z, excluding dynamic gestures J and Z (a total of 24 classes representing different letters). This dataset includes 34,627 cases. In this dataset, 85% of the data were used for training and 15% were used for testing.

### 3.2. Preprocessing

The original image size is 250 × 250 pixels. The image was re-sized to 229 × 229 for TSM-AlexNet and TSM-LeNet and 226 × 226 for TSM-ResNet18 and TSM-ResNet50. Gray normalization was performed. Normalized data have the same mean and variance to reduce effects of the environment for correct recognition.

Data augmentation can improve the classification accuracy of the CNN algorithms [32] by extending image data. In this paper, three augmentation methods of rotation, scaling, and translation were used to generate new training sets. The rotation operation was used to rotate the image in the clockwise direction by an angle between 0 and 360 degrees and to fill the pixel in the lost pixel area of the image. The scaling operation was used to magnify or reduce the image. The translation was conducted by either translating the image in a horizontal or vertical direction. The rotation of 45 degrees, scaling magnification of 10%, horizontal translation by 10%, and vertical translation by 10% were used for image augmentation.

### 3.3. Proposed TSM-CNN

**Figure 3:**
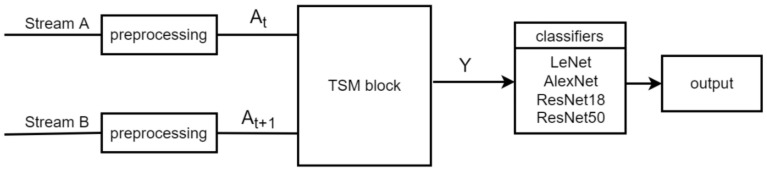
The overview of TSM-CNN.

The proposed TSM-CNN system was composed of preprocessing, the TSM block, and classifiers as shown in Figure 3. Two consecutive images for the dynamic gesture or two identical images for the static gesture were used as inputs of streams A and B; resizing, transformation, and augmentation were carried out in the preprocessing stage. The feature map Y was obtained by addition and concatenation in the TSM block. Finally, a classifier was used to classify images. The TSM-CNN model with the highest performance scores depending on three concatenation methods was selected as the definitive recognition model for ASL recognition.

#### TSM Block

**Figure 4:**
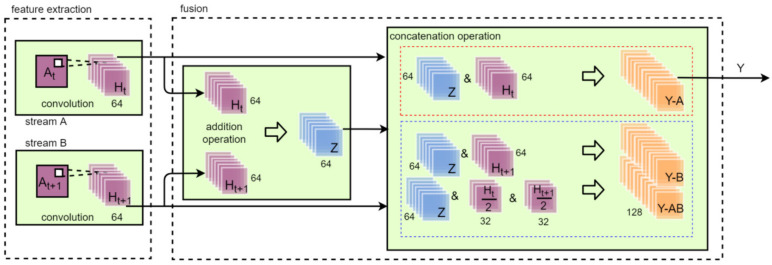
TSM structure.

The proposed TSM comprised feature extraction and fusion as shown in Figure 4. The goal of TSM is to enhance correlation information expression between two consecutive images. The accuracy of dynamic image recognition always relies on correlation information. Therefore, TSM can improve the accuracy of dynamic gesture recognition by considering two consecutive images. The convolution kernel size of TSM was 3 × 3 and the stride was 1. Three different kernel sizes (3 × 3, 5 × 5, and 7 × 7) were used as a comparison group to select the suitable kernel size. In the feature extraction part, feature maps $H_{t1}$ and $H_{t2}$ were obtained after the convolution. The number of channels was 64 in $H_{t1}$ and $H_{t2}$.

The addition and concatenation operator were used in the fusion block. The mixed feature map *Z* was obtained by addition of $H_{t}$ and $H_{t+1}$. The addition operation was used to add two consecutive feature maps at the pixel level. The number of channels in *Z* was 64. The number of channels in Y-A, Y-B, Y-AB, and *Y* was 128. The addition operator calculated mixed feature maps for dynamic gestures and increased the image contrast for static gestures. Because the value range of each pixel in an image was from 0 to 255, the addition operation made the background brighter. However, the dark area of the gesture was not greatly affected. This made the contrast of static gesture images significantly enhanced.

The concatenation operation in the fusion block was used to obtain fusion feature maps between images without losing the original image data so that the recognition accuracy could be improved. Feature maps Y-A, Y-B, and Y-AB were obtained by the concatenation operation of *Z* with $H_{t}$, *Z* with $H_{t+1}$, and *Z* with half $H_{t}$ and half $H_{t+1}$, respectively. They were named as TSMA, TSMB, and TSMAB for three feature maps Y-A, Y-B, and Y-AB, respectively. The output of TSM was used as the input of the CNN classifier. The results of TSMA-ResNet50, TSMA-ResNet50, and TSMA-ResNet50 were compared to choose the most suitable feature map as *Y*.

The feature extraction in TSM is calculated with the following equation:

(1) $$H_{t}=\sum_{j}\sum_{k}W_{i}[j,k]A_{t}[a-j,a-k]$$

In Equation (1), W is the kernel matrix; $A_{t}$ and $A_{t+1}$ are the input matrixes; $H_{t}$ and $H_{t+1}$ are feature maps from different streams after convolution; *i* represents the number of streams; *j* and *k* represent the index of the row and column in the kernel, respectively. *a* is the length and width of the input data because the image in TSM has the same length and width. The feature map *Z* in TSM is calculated with Equation (2):

(2) $$\begin{array}{c} Z=H_{t}+H_{t+1}\\ =\sum_{j}\sum_{k}W_{A}[j,k]A_{t}[a-j,a-k]+W_{B}[j,k]A_{t+1}[a-j,a-k] \end{array}$$

The information between two consecutive dynamic images is extracted in the addition operation. The concatenation operation aims to retain the original information, which is defined as Equation (3), where *c* is the total number of channels, *l* is the index of channels, and *&* means the concatenate operator. The feature map *Y* in *TSM* is calculated with Equation (3):

(3) $$Y=Output_{TSM}=\sum_{l=1}^{c-l}Z_{i}\&\sum_{c-l}^{c}H_{t}$$

CNN models were used for the final classification after TSM. The results were compared to select the best model for sign language recognition.

**Figure 5:**
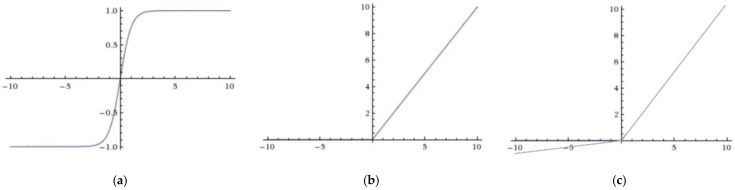
Different activation functions. (**a**) Tanh, (**b**) ReLu, (**c**) Leaky ReLu.

Table 1 shows the architecture of the TSM. The 3 × 3 kernel size was selected for the convolution layer, and the feature map of Z concatenated with $H_{t}$ was chosen as the suitable feature map Y. The TSM was the pre-operation of deep learning classifiers to expand the diversity of features, so the activation function was selected for TSM from Tanh, ReLu, and Leaky ReLu to enhance the feature expression ability [33]. The Tanh function caused the vanishing gradient problem when the data were too large or too small. The ReLu function solved this problem better as shown in Figure 5b, but the negative axis for ReLu brought the dead neuron problem, causing the gradient to not propagate. In Figure 5c, the negative axis for Leaky ReLu compared to the ReLu function had a leak value, so the dead neuron problem was alleviated.

In other research [34], Leaky ReLu was applied to deep learning and showed excellent performance, so this paper chose Leaky ReLu for the activation function of the convolutional layer in TSM.

### 3.4. Structure of Classifiers

Some single-stream CNN models were introduced to build TSM-CNN models, including TSM-LeNet, TSM-AlexNet, TSM-ResNet18, and TSM-ResNet50.

#### 3.4.1. LeNet and AlexNet

LeNet [6] is the cornerstone of CNN development. It introduces the concept of convolution into the neural network, bringing better feature extraction ability in the recognition task. The gradient descent updates model parameters in the backpropagation. The convolution-pooling-fully connected framework to better obtain representative features through training has laid the foundation for the development of CNN. The model has been successfully applied to the handwritten digit classification task, achieving high accuracy.

Krizhevsky et al. [35] built the AlexNet model to defeat SVM and gain first place in the 2012 image classification algorithm competition, bringing CNN to the mainstream recognition method. AlexNet inherits the basic structure from LeNet. The specific framework is shown in Figure 5. There are eight main layers in AlexNet, including three convolutional layers, three pooling layers, and two fully connected layers. Each layer of convolution has an activation function. The pooling layer is used for down-sampling to reduce the image size for easy calculation. The full connection layer is used for final recognition. In addition, the dropout layer is added after the last convolution-pooling structure to prevent overfitting.

The modified LeNet and AlexNet structures in this paper are shown in Table 2. The basic framework, ReLu in convolution and Softmax for the multi-class classification task, follows the structure of LeNet and AlexNet [6,35] that has been proven effective in many image recognition tasks. In addition, the Batch Normalization [36] is appended after each convolution layer to mitigate the effect of unstable gradients within a neural network through the introduction of an additional layer that performs operations on the inputs from the previous layer.

#### 3.4.2. ResNet

**Figure 6:**
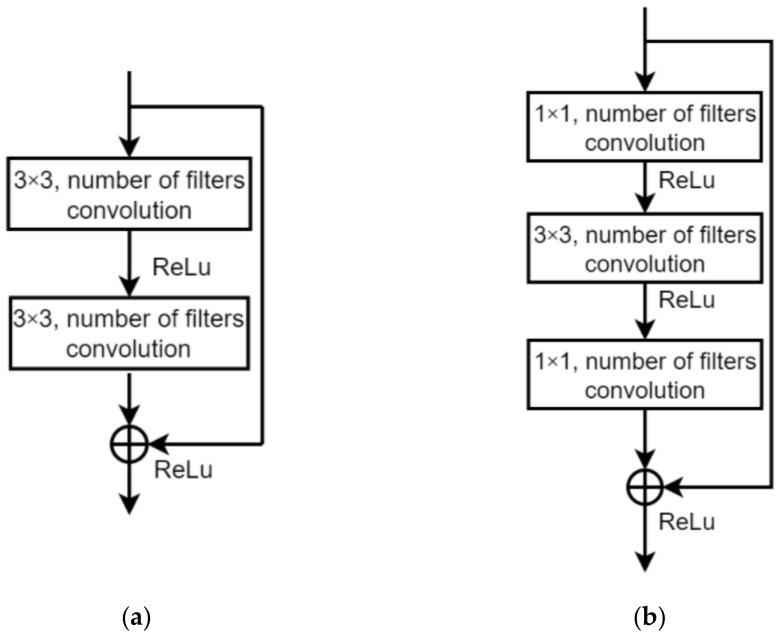
Residual module structure. (**a**) ResNet18, (**b**) ResNet50.

ResNet was proposed to solve the problem of model convergence difficulty in the last stage of training in CNN [28]. ResNet uses a new structure called the residual module, which is accessed in the CNN to train the model according to the difference between input and output in the current layer and previous layer, respectively. The application of the residual module effectively improves the recognition accuracy. The traditional neural network only learns the mapping from the input image to the output label, not including the middle information between layers in CNN. However, ResNet considers middle information in the training process to achieve better recognition accuracy as shown in Figure 6a.

The most significant difference between ResNet18 and ResNet50 is the use of the bottleneck structure. The key to the bottleneck structure is application of the 1 × 1 convolution. The 1 × 1 convolution takes more nonlinear mappings and maintains the original feature map size as shown in Figure 6b. Compared with other sizes of convolution kernels, 1 × 1 convolution can significantly reduce computational complexity. There are a total of eight identical residual modules used in ResNet18 to increase data computability. In ResNet50, these eight residual modules with 1 × 1 convolution are applied to gain more feature extraction improvement than in ResNet18.

The ResNet18 and ResNet50 in this paper are shown in Table 3. The Residual module (a) and Residual module (b) are the Residual modules of ResNet18 and ResNet50, respectively. The Softmax for the multi-class classification task follows the structure of ResNet18 and ResNet50 that has been proven effective in many image recognition tasks [37].

### 3.5. Evaluation Method

Performances of different CNNs for the testing dataset were evaluated and compared using accuracy, recall, precision, and F1 score evaluation methods.

(4) $$\mathrm{Accuracy}=\frac{\mathrm{TP}+\mathrm{TN}}{\mathrm{TP}+\mathrm{TN}+\mathrm{FP}+\mathrm{FN}}$$

(5) $$\mathrm{Precision}=\frac{\mathrm{TP}}{\mathrm{TP}+\mathrm{FP}}$$

(6) $$\mathrm{Recall}=\frac{\mathrm{TP}}{\mathrm{TP}+\mathrm{FN}}$$

(7) $$F1\mathrm{score}=\frac{2\mathrm{Precision}\times \mathrm{Recall}}{\mathrm{Precision}+\mathrm{Recall}}$$

where TP is True Positive, FN is False Negative, FP is False Positive, and TN is True Negative. These four evaluation parameters were used to measure the effectiveness of the model.

## 4. Experiments

### 4.1. Implementation Details

All experiments were performed on an Intel Quad Core i7 CPU and Tesla-K80 Nvidia graphics card. We implemented our code using Python. Opencv2.4.1 was used for computer vision operations of data processing. TensorFlow 1.15 was used for the deep learning CNN model.

### 4.2. Results of TSM Method

**Figure 7:**
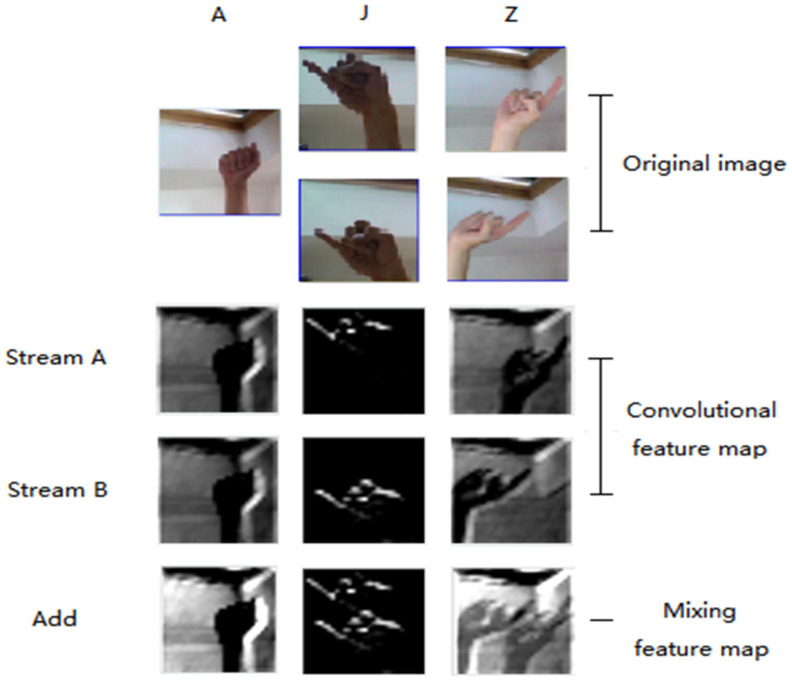
Different Feature maps.

Figure 7 shows images at outputs of feature extraction and the addition operation. J and Z were dynamic gestures and A was the static gesture. Original images were input to stream A and stream B. The convolutional feature maps were generated after convolution processing in two streams. After the addition layer, the feature contrast was enhanced for static gestures and motion features were preserved for dynamic gestures.

### 4.3. Comparison of Results

Table 4 shows accuracy results for the three kernel sizes. TSM-ResNet50 was used to choose the best kernel size for sign language feature extraction in the TSM. The 3 × 3 convolution kernel for feature extraction had the highest accuracy.

Table 5 shows accuracies of the three models depending on the concatenation method. They showed almost the same results, with TSMA-ResNet50 having a slightly higher accuracy. TSMA was selected in a concatenation operation for feature map Y. The TSMA-ResNet50 was named as TSM-ResNet50 for sign language recognition in this paper.

Table 6 shows the results of TSM-CNNs for MNIST and ASL datasets. Models with a TSM block had better performance than those without a TSM block. The TSM method helped CNN extract correlation features of dynamic gesture images in sign language. According to the time for recognition of one time from the MNIST and ASL test dataset, the calculation time of each model was not affected much after using the TSM method. The test time for recognition of one time in the TSM-ResNet18 and TSM-ResNet50 models was less than 0.5 s and also satisfied the real-time recognition requirements as a classification part. The accuracy of each CNN with the application of TSM also increased. Thus, the TSM method is relatively efficient, which improves the neural network recognition performance.

From the recognition results of the MNIST dataset with only static gestures, the use of the TSM method also improved the accuracy. The addition operation in TSM helped static gestures achieve a clearer expression. The TSM-ResNet50 achieved the best result in both MNIST and ASL Alphabet datasets. Thus, this model was chosen for the sign language recognition model in this paper. Evaluation results showed that the processing results of the models were effective and creditable. TSM-CNN minimized the error rate in the recognition of dynamic gestures J and Z as shown in Table 7.

**Figure 8:**
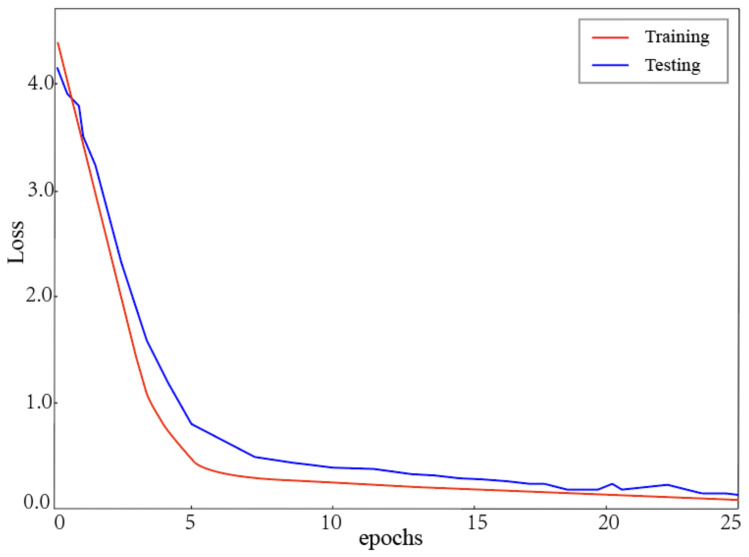
Training and test loss of TSM-ResNet50 depending on the epoch.

The results showed that the recognition accuracy was increased by the application of TSM. The TSM retained the original information and the correlation information to help the CNN model recognize dynamic gestures more accurately. The addition operation enabled better expression of correlation features between current and previous images. The concatenation operation prevented the loss of convolutional feature maps. Figure 8 shows loss curves vs. epochs for TSM-ResNet50. Training loss and test loss converged. The gap between them was minimal. TSM-ResNet50 had a good performance in ASL recognition.

## 5. Discussion

ASL recognition is a branch of SL recognition, as an auxiliary language mainly used for spelling correction, the spelling of people’s names, and book titles. ASL is used relatively infrequently among deaf people but is indispensable. Compared with other SL categories, the ASL expression is relatively simple. However, the high similarity of some gestures in ASL challenges accurate recognition. There is currently a lack of more efficient techniques with lower model complexity for ASL recognition, so the TSM-CNN method is proposed in this paper. TSM-ResNet50 is selected for ASL recognition finally.

Table 8 shows results of the comparison with some previous works. The proposed work achieves almost the highest accuracy in 29 classes of ASL recognition. The mobileNet of Alashhab et al. classify only five classes of gestures. Thus, the accuracy is high. The RNN-based system [38] fuses four deep RNN models to study the sequences, and a deep learning model consisting of BLSTM in a 3D-ResNet enhances series learning for sign language recognition. However, due to the high complexity of those models, they cannot show their superiority in the ASL alphabet recognition. For this supplementary and indispensable simple sign language, combining sequence information and original image information through the proposed TSM method with the powerful recognition ability of CNN reduces the complexity of the model and low computing consumption, and obtain relatively accurate recognition results. The self-mutual distillation learning-based system [39] yields a label for each time step concerning the continuous words. The 3D ConvNet with the BiLSTM system [40] is used for data extraction and enhancement of time series information to increase the model performance. These two methods exhibit excellent recognition performance in datasets different from ASL. However, as the expression of ASL Alphabet is not a complex expression that strongly depends on time and continuous actions or gestures, it is difficult for these methods to show excellent performance for ASL Alphabet dataset recognition in this paper. The highly time-series-dependent and complex model also makes it difficult to achieve outstanding performance in ASL. Our model is built for better ASL recognition with low model complexity, and the CNN-based method achieves better expression for the feature information of the gestures. Our model is designed to focus on the ASL alphabet dataset, so it has a simple structure, low time consumption, and high accuracy, but it is not appropriate to recognize other ALS languages with highly time-series-dependent and complex movements, which is the limitation and disadvantage of our model.

With the 2D-CNN with the joints encoding [41] method with high-hands-information-capture-hardware requirements, our model achieves better performance at a lower cost with only a camera. An LSTM method for ASL recognition [42] with four different sequential shows excellent performance in dynamic images, but lower recognition performance in static images that do not rely on sequences, so using CNN as the final classification method with the proposed TSM block is more suitable for ASL recognition.

The TSM method proposed in this paper makes up for shortcomings of traditional single-stream CNN [43,44,45,46,47] in poor processing dynamic gesture data, making CNN more flexible in processing image classification problems and higher accuracy. Our proposed TSM method simultaneously improves the feature extraction ability of dynamic and static gestures with higher recognition performance due to the addition and concatenation operations in TSM that enable features to be expressed more abundantly without losing information.

The proposed TSM-ResNet50 model demonstrates its feasibility as a recognition module in the RGB capture-based translation tool, and an actual application of our model is to help hearing-impaired people better communicate when they need to use the recognition system in name spelling, book spelling, and letter correction.

## 6. Conclusions and Future Work

Deep learning technology has achieved great success in speech recognition, image classification, target detection, and other fields. The application of deep learning models developed for various computer vision fields has been used in our daily life.

In this paper, a TSM method was proposed for CNN performance improvement. The TSM-CNN system was composed of preprocessing, the TSM block, and CNN classifiers. Two consecutive images for dynamic gestures were used as inputs of streams A and B. Models of LeNet [6], AlexNet [7], ResNet18 [8], and ResNet50 [9] with or without TSM were compared to evaluate the performance of the TSM. Experimental results showed that application of TSM improved the feature capture ability for dynamic gestures. An addition operation was performed in the fusion step to obtain correlation information between current and previous images, which increased the accuracy of recognition. Therefore, the resulting feature vector from TSM had a stronger discernibility. The experimental results also showed that the TSM-ResNet50 model had better performance than several other CNN models.

In the future, a real-time, high-accuracy, and relatively low-cost sign language recognition system will be developed for recognizing dynamic gestures or videos in other fields and a sign language recognition system for hearing-impaired people.

## Figures and Tables

**Table 1:** The architecture of the TSM.

TSM Architecture	
kernel stream A	3 × 3 × 64 (kernel size)
kernel stream B	3 × 3 × 64 (kernel size)
$\mathrm{Addition}\;\mathrm{operation}:\;Z=H_{t}$ $+H_{t+1}$	64 (channel)
$\mathrm{Concatenation}\;\mathrm{operation}:\;Y=H_{t}$ *& Z*	128 (channel)

**Table 2:** The architecture of the LeNet and AlexNet.

CNN Model	Architecture	Kernel Size	Output Shape
LeNet	Input	-	227 × 227 × 128
	Convolution_1	5 × 5, 128, stride = 2	75 × 75 × 128
	Maxpooling_1	2 × 2, stride = 2	37 × 37 × 128
	Convolution_2	5 × 5, 256, stride = 2	11 × 11 × 256
	Maxpooling_2	2 × 2, stride = 2	5 × 5 × 256
	Flatten	-	6400
	Fully connection_1	-	1280
	Fully connection_2	-	256
	Output	-	29
AlexNet	Input	-	227 × 227 × 128
	Convolution_1	11 × 11, 128, stride = 4	55 × 55 × 128
	Maxpooling_1	3 × 3, stride = 2	27 × 27 × 128
	Convolution_2	5 × 5, 256, stride = 1	27 × 27 × 256
	Maxpooling_2	3 × 3, stride = 2	13 × 13 × 256
	Convolution_3	3 × 3, 384, stride = 1	13 × 13 × 384
	Convolution_4	3 × 3, 384, stride = 1	13 × 13 × 384
	Convolution_5	3 × 3, 256, stride = 1	13 × 13 × 256
	Maxpooling_5	3 × 3, stride = 2	6 × 6 × 256
	Flatten	-	9216
	Fully connection_1	-	4096
	Fully connection_2	-	4096
	Output	-	29

**Table 3:** The architecture of the ResNet18 and ResNet50.

CNN Model	Architecture	Kernel Size	Output Shape
ResNet18	Input	-	224 × 224 × 128
	Convolution_1	7 × 7, 128, stride = 2	112 × 112 × 128
	Maxpooling_1	3 × 3, stride = 2	56 × 56 × 128
	Residual module(a)_1 ×2	3 × 3, 128, stride = 1 3 × 3 × 128, stride = 1	56 × 56 × 128
	Residual module(a)_2 ×1	3 × 3, 256, stride = 2 3 × 3 × 256, stride = 1	28 × 28 × 256
	Residual module(a)_3 ×1	3 × 3, 256, stride = 1 3 × 3 × 256, stride = 1	28 × 28 × 256
	Residual module(a)_4 ×1	3 × 3, 256, stride = 2 3 × 3 × 256, stride = 1	14 × 14 × 256
	Residual module(a)_5 ×1	3 × 3, 256, stride = 1 3 × 3 × 256, stride = 1	14 × 14 × 256
	Residual module(a)_6 ×1	3 × 3, 512, stride = 2 3 × 3 × 512, stride = 1	7 × 7 × 512
	Residual module(a)_7 ×1	3 × 3, 512, stride = 1 3 × 3 × 512, stride = 1	7 × 7 × 512
	Avgpooling_1	7 × 7	1 × 1 × 512
	Fully connection_1	-	512
	Output	-	29
ResNet50	Input	-	224 × 224 × 128
	Convolution_1	7 × 7, 128, stride = 2	112 × 112 × 128
	Maxpooling_1	3 × 3, stride = 2	56 × 56 × 128
	Residual module(b)_1 ×1	1 × 1, 128, stride = 2 3 × 3 × 128, stride = 1 1 × 1 × 256, stride = 1	56 × 56 × 256
	Residual module(b)_2 ×2	1 × 1, 128, stride = 1 3 × 3 × 128, stride = 1 1 × 1 × 256, stride = 1	56 × 56 × 256
	Residual module(b)_3 ×1	1 × 1, 256, stride = 2 3 × 3 × 256, stride = 1 1 × 1 × 512, stride = 1	28 × 28 × 512
	Residual module(b)_4 ×3	1 × 1, 256, stride = 1 3 × 3 × 256, stride = 1 1 × 1 × 512, stride = 1	28 × 28 × 512
	Residual module(b)_5 ×1	1 × 1, 512, stride = 2 3 × 3 × 512, stride = 1 1 × 1 × 1024, stride = 1	14 × 14 × 1024
	Residual module(b)_6 ×5	1 × 1, 512, stride = 1 3 × 3 × 512, stride = 1 1 × 1 × 1024, stride = 1	14 × 14 × 1024
	Residual module(b)_7 ×1	1 × 1, 1024, stride = 2 3 × 3 × 1024, stride = 1 1 × 1 × 2048, stride = 1	7 × 7 × 2048
	Residual module(b)_8 ×2	1 × 1, 1024, stride = 1 3 × 3 × 1024, stride = 1 1 × 1 × 2048, stride = 1	7 × 7 × 2048
	Avgpooling_1	7 × 7	1 × 1 × 2048
	Fully connection_1	-	2048
	Output	-	29

**Table 4:** Feature Extraction Kernel Size Selection of TSM-ResNet-50 in ASL Alphabet Dataset.

Kernel Size	Accuracy
3 × 3	97.57%
5 × 5	97.47%
7 × 7	97.42%

**Table 5:** Accuracy of TSM-ResNet-50 for ASL Alphabet Dataset.

Structure	Accuracy
TSMA-ResNet50	97.57%
TSMB-ResNet50	97.49%
TSMAB-ResNet50	97.51%

**Table 6:** Results Comparison of TSM-CNNs.

Structure	MNIST Dataset					ASL Alphabet Dataset				
	Accuracy	Precision	Recall	F1-Score	Time	Accuracy	Precision	Recall	F1-Score	Time
LeNet	89.31%	88.72%	88.23%	88.93%	1.1 ms	88.43%	87.68%	87.35%	87.76%	1.1 ms
TSM-LeNet	90.42%	91.58%	91.76%	91.16%	1.3 ms	89.18%	91.35%	91.17%	91.35%	1.4 ms
AlexNet	94.74%	89.95%	89.38%	89.24%	5.0 ms	93.64%	88.46%	87.88%	87.92%	4.8 ms
TSM-AlexNet	94.96%	93.54%	93.45%	93.75%	5.2 ms	94.07%	93.22%	93.52%	92.91%	5.1 ms
ResNet18	98.13%	94.16%	94.23%	94.11%	11.1 ms	96.97%	93.77%	94.38%	94.17%	11.4 ms
TSM-ResNet18	98.36%	94.25%	94.56%	94.34%	11.4 ms	97.11%	93.98%	93.66%	93.54%	11.7 ms
ResNet50	98.88%	94.37%	94.27%	94.30%	25.6 ms	97.41%	94.01%	93.56%	93.88%	25.1 ms
TSM-ResNet50	99.09%	94.48%	94.56%	94.52%	25.8 ms	97.57%	94.36%	94.07%	94.06%	25.5 ms

**Table 7:** Results of Comparing J and Z in ASL Dataset.

Structure	Error Rate	
	J	Z
LeNet	11.61%	11.09%
TSM + LeNet	10.81%	10.13%
AlexNet	7.93%	8.15%
TSM + AlexNet	7.66%	7.78%
ResNet18	5.18%	4.93%
TSM + ResNet18	4.86%	4.67%
ResNet50	4.72%	4.59%
TSM + ResNet50	4.51%	4.36%

**Table 8:** Comparison of Our Work with Previous Works.

Authors	Works	Accuracy
Das, A. et.al., 2018 [43]	Transfer learning using Inceptionv3 on custom dataset	90.0%
Alashhab, S. et. al., 2018 [44]	Transfer learning on multiple architectures in VGGNet, ResNet, etc., on 5 custom classes of hand gestures	99.45%
Kania, K. et. al., 2018 [45]	Transfer learning using Wide Residual Networks with data augmentation on ASL alphabet	93.3%
Garcia and Viesca, 2016 [46]	CNN on 24 ASL Alphabet with GoogLeNet transfer learning	70%
Bousbai, K. and Merah, M. 2019 [47]	Compare custom CNN model and transfer learning using MobileNetV2 on ASLs	97.06%
QingGao et al., 2019 [29]	2S-CNN model on ASL dataset	92.08%
Borg et.al., 2020 [37]	RNN-based system for RWTH-Phoenix Weather dataset of sign language recognition	97.19%
Li et.al., 2021 [41]	2D-CNN with joints encoding hand gesture recognition for 14-class ASL alphabet	96.31%
Hao et al., 2021 [38]	Self-mutual distillation learning for sign language recognition	80%
Adaloglou et al., 2022 [40]	Inflated 3D ConvNet with BLSTM for sign language recognition	89.74%
Kothadiya et. al., 2022 [42]	Four different sequences of LSTM and GRU for ASL recognition	95.3%
Proposed Work	TSM-ResNet50 on 29 classes of ASL Alphabet	97.57%

## Data Availability

The datasets used in this paper are come from Kaggle’s website [30,31].
